# The effectiveness of physeal bar resection with or without Hemi-Epiphysiodesis to treat partial growth arrest

**DOI:** 10.1186/s12891-023-06167-6

**Published:** 2023-01-30

**Authors:** Han Xiao, Miao Li, Guanghui Zhu, Qian Tan, Weihua Ye, Jiangyan Wu, Haibo Mei, An Yan

**Affiliations:** 1 Department of Pediatric Orthopaedics, Hunan Children’s Hospital, No 86 Ziyuan Road, Yuhua District, Hunan Province 410007 Changsha City, China; 2 The Pediatric Academy of University of South China, 410007 Changsha, Hunan China

**Keywords:** Partial physeal growth arrest, Physeal bar resection, Hemi-Epiphysiodesis technique, Angular deformity, LLD

## Abstract

**Purpose:**

To evaluate the outcomes of distal femoral, proximal tibial, and distal tibial physeal bar resection combined with or without the Hemi-Epiphysiodesis procedure and provide a better understanding of the application of physeal bar resection combined with Hemi-Epiphysiodesis procedure in the treatment of physeal bar growth arrest.

**Methods:**

We retrospectively reviewed the patients who suffered physeal bar and underwent physeal bar resection with or without the Hemi-Epiphysiodesis technique during 2010–2020. All were followed up for at least 2 years or to maturity. A modified mapping method was used to determine the area of a physeal bar by CT data. The aLDFA, aMPTA, aLDTA, MAD, and LLD were measured to assess the deformity of the lower limb.

**Results:**

In total, 19 patients were included in this study. The average age was 8.9 years (range 4.4 to 13.3 years old). During the follow-up, 4 (21.1%) patients had an angular change < 5°; 12 (63.2%) patients had angular deformity improvement > 5° averaging 10.0° (range 5.3° to 23.2°), and 3 (15.8%) patients had improvement of the angular deformity averaging 16.8° (range 7.4° to 27.1°). Eleven patients (57.9%) had significant MAD improvement. After surgery, we found that 7 (36.8%) patients had an LLD change of < 5 mm and were considered unchanged. Only 2 (15%) patients had an LLD improvement > 5 mm averaging 1.0 cm (range 0.7 to 1.3 cm), and 7 (36.8%) patients had increasing of LLD > 5 mm averaging 1.3 cm (range 0.5 to 2.5 cm). There were no postoperative fractures, infections, or intraoperative complications such as neurovascular injury.

**Conclusion:**

Physeal bar resection combined with Hemi-epiphysiodesis is helpful for partial epiphysis growth arrest. Without statistically verifying, we still believe that patients with limited growth ability could benefit more from physeal bar resection combined with Hemi-epiphysiodesis.

## Introduction

Partial physeal growth arrest is an infrequent complication of epiphysis injury. A bone bridge or physeal bar forms and can cause severe angular and length growth abnormality of the extremity [1, 2]. Any physeal damage resulting from fracture, infection, tumor, irradiation, or iatrogenic insertion of metal across the epiphysis, can lead to the formation of a physeal bar and tethers bone growth [3]. Among them, physeal bar formation secondary to trauma is most common in clinics. Reports showed that 18–30% of pediatric fractures were associated with physeal injuries, and up to 10% of these injuries would have growth arrest [4].

Treating partial physeal growth arrest is a challenge to pediatric surgeons since the associated progressive angular deformity or shortening may have a devastating effect on limb alignment, adjacent joint function, and longevity [5]. So far, physeal bar resection has been proven a promising method to restore the growth ability of the growth plate [6]. There is a consensus that patients with at least 2 years or 2 cm of growth remaining and less than 50% physeal area may benefit from the physeal bar resection responsible for the arrest [7, 8]. Many surgical techniques have been described for physeal bar resection, including fluoroscopy, arthroscopy, and recently navigation-assisted physeal bar excision [9–11].

Limb realignment or limb lengthening should be considered for patients with concomitant angular deformity or leg length discrepancy (LLD). Kim et al. applied complete transverse osteotomy of the metaphysis near the bar with a direct vertical approach, allowing visualization of a centrally located bar and the adjacent healthy physis. Ilizarov external fixation can also correct this associated angular deformity and permit limb lengthening [12]. Still, this procedure is more invasive, and the indications for corrective osteotomy at the time of bridge resection are controversial. In recent years, Hemi-Epiphysiodesis with tension-band plates has gained interest as a less invasive procedure for treating angular deformities in skeletally immature patients [10]. However, how to appropriately use the growth modulation technique to correct the angular or LLD deformity is controversial.

In this study, we aim to evaluate the outcomes of distal femoral, proximal tibial, and distal tibial physeal bar resection combined with or without Hemi-Epiphysiodesis procedure and provide a better understanding of the application of physeal bar resection combined with Hemi-Epiphysiodesis procedure in the treatment of partial physeal growth arrest.

## Methods and materials

### Patients

After institutional review board approval, we retrospectively reviewed the patients who suffered partial growth arrest and underwent physeal bar resection by Langenskiöld’s procedure during 2010–2020. Physeal growth arrest was diagnosed by clinical and computed tomography evidence. Patients who didn’t follow up for at least two years were excluded. In total, 19 patients were included in this study. The average age was 8.9 ± 2.5 years (range 4.4 to 13.3). The percent of the bar area was 15.2 ± 6.7% (range, 6.6 to 28.6). Seven patients received physeal bar resection, while 12 received physeal bar resection combined with the Hemi-Epiphysiodesis technique (Hemi-Epiphysiodesis). Twelve patients were followed to maturity. The demographic characteristics of the 19 patients are shown in Table 1.

**Table 1:** Patient demographics

Case	Gender	Age at surgery (yrs)	Location	Percent involved (%)	Type	Growth guided technique	Period of follow-up (month)	To maturity
1	M	11.9	Distal femur (R)	7	Peripheral	No	47	Yes
2	F	4.4	Distal femur (R)	26	Central	No	56	No
3	M	9.8	Distal femur (R)	20	Peripheral	No	62	Yes
4	F	7.1	Distal tibia (R)	7	Central	No	54	No
5	F	7.5	Distal tibia (R)	12	Mixed	No	38	No
6	F	5.8	Distal tibia (L)	13	Mixed	No	24	No
7	M	6.9	Distal femur (L)	23	Central	No	62	No
8	M	7.9	Distal femur (R)	12	Peripheral	Yes	98	Yes
9	M	7.6	Distal femur (L)	8	Peripheral	Yes	85	Yes
10	M	9.5	Distal femur (L)	14	Central	Yes	76	Yes
11	F	12.2	Distal femur (R)	17	Central	Yes	48	Yes
12	M	9.6	Distal femur (L)	11	Peripheral	Yes	28	Yes
13	F	8.8	Distal femur (R)	9	Peripheral	Yes	58	Yes
14	F	12	Distal femur (L)	18	Mixed	Yes	30	Yes
15	F	6.8	Distal femur (R)	10	Central	Yes	29	No
16	M	13.3	Proximal tibia (L)	10	Mixed	Yes	25	Yes
17	F	7.6	Distal femur (R)	19	Peripheral	Yes	72	Yes
18	M	8.7	Distal femur (L)	23	Mixed	Yes	68	Yes
19	M	12.4	Distal femur (R)	29	Peripheral	Yes	33	Yes

*M* represent for Male, *F* represent for Female, *L *left, *R *right

### Radiographic imaging

**Fig. 1:**
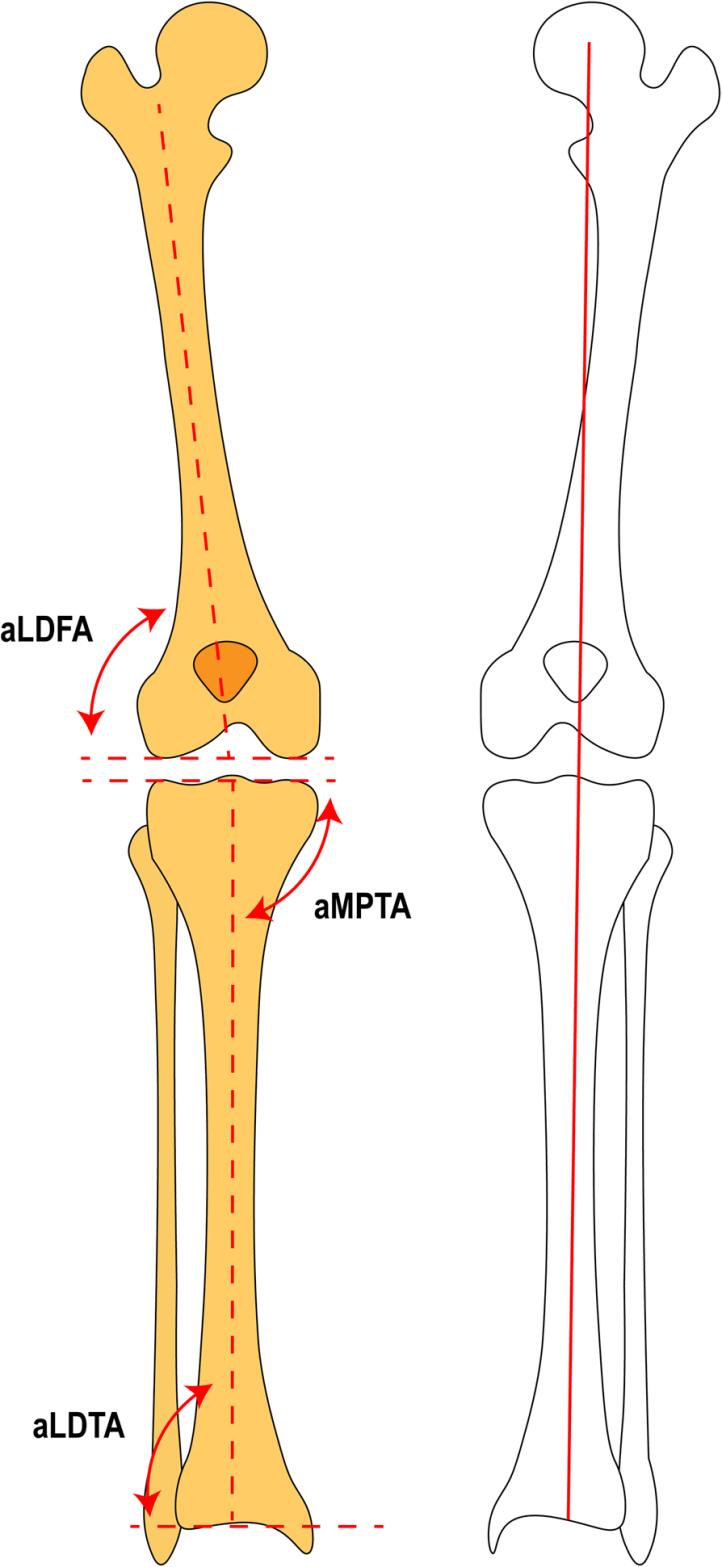
The schematic measurement diagram of the aLDFA, aMPTA, aLDTA, and LLD

A modified mapping method was used to determine the area of a physeal bar by CT data. In brief, we used the Mimics 19.0 software (Materialise, Leuven, Belgium) to reconstruct the knee. Then, the epiphysis was isolated. The projected area of both the epiphysis and bony bar in the epiphysis on a plane perpendicular to the femoral anatomical axis was calculated. For assessment of angular deformity, mechanical axis deviation (MAD), and LLD, standard entire lower limb radiographs were taken in the anteroposterior views (Fig. 1). The preoperative and postoperative aLDFA, aMPTA, and aLDTA were measured to assess the corrective angular deformity. The LLD was determined by the discrepancy between the femoral head center and distal tibial center [13]. The angular deformity in the coronal plane, MAD, and LLD were measured by two pediatric surgeons with the PACS system (1.0.0.1, PowerBuilder Enterprise Sesires, USA).

### Surgical techniques

**Fig. 2:**
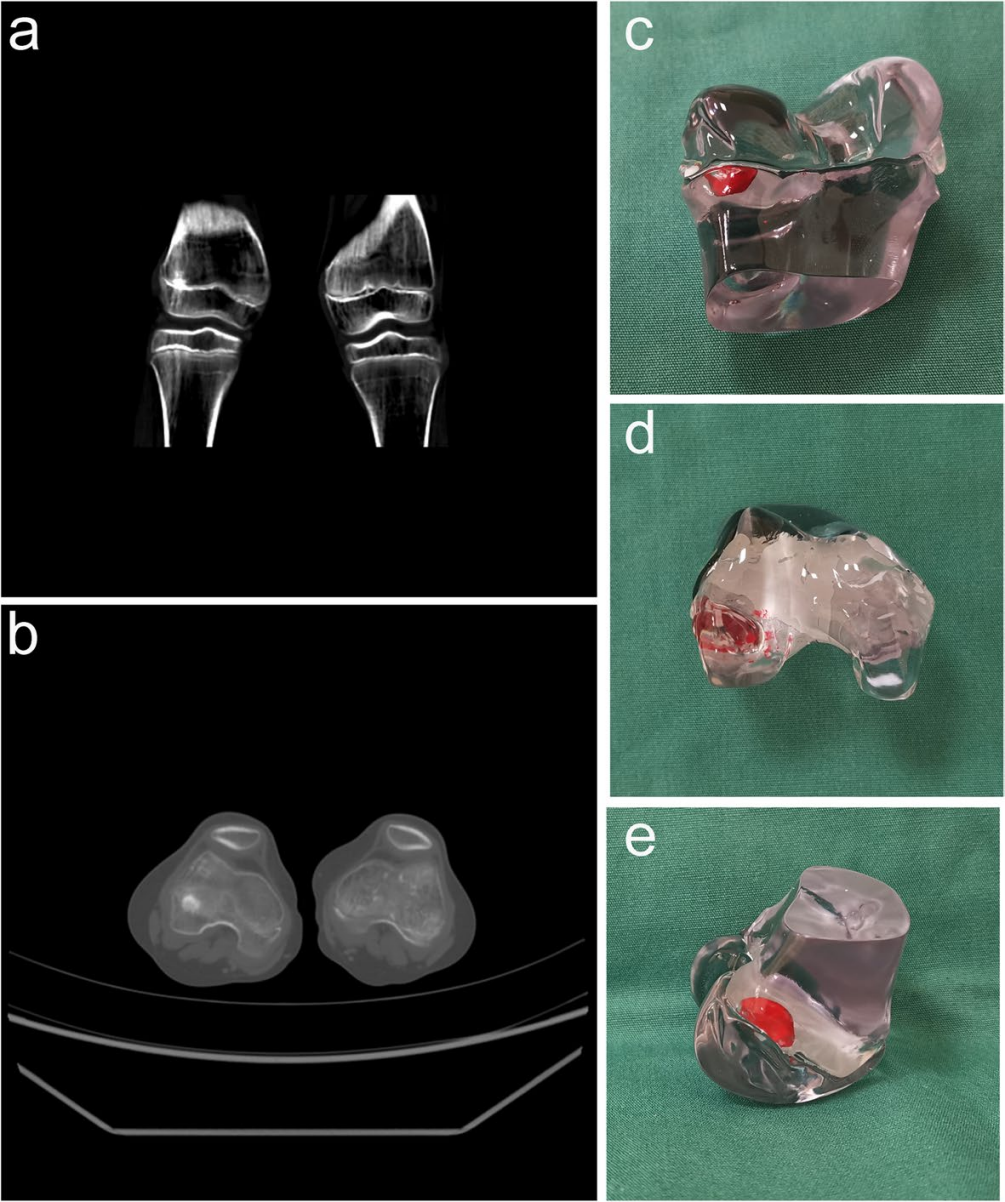
The visualization of the physeal bar in CT and 3D printing model. The coronary view (**a**) and horizontal view (**b**) in CT; The posterior-anterior view (**c**), up-down view (**d**), and lateral-medial view (**e**) of the model. The red part represents the physeal bar

**Fig. 3:**
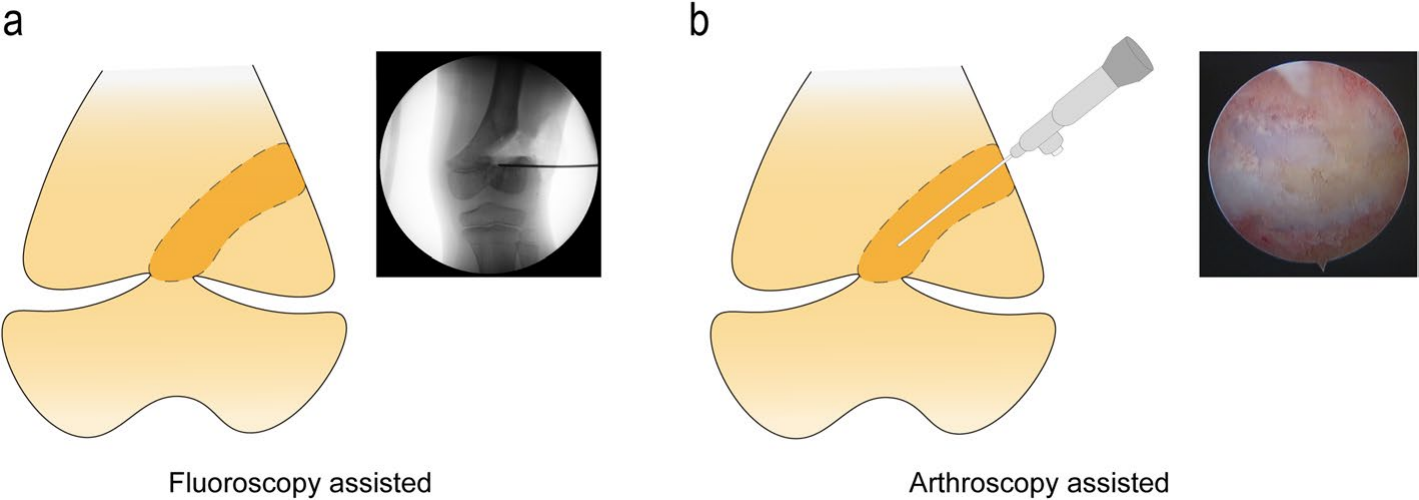
The surgical procedure diagram with fluoroscopy (**a**) and arthroscopy lamp (**b**)

The physeal bar resection was performed based on a previous report. A full-size distal femur model showing the location of the physeal bar was printed to assist the surgeon during the procedure (Fig. 2). After administering general anesthesia, the patient was supine on the operating table with the tourniquet applied to the proximal end of the lower extremity. The peripheral bar was removed through a direct approach, while the central physeal bar was typically resected through the metaphysis window adjacent to the physeal bar. With fluoroscopic guidance, the physeal bar was carefully removed by a motorized burr. We used the arthroscopic lamp to remove the physeal bar, and normal epiphysis was encountered at the last surgical step (Fig. 3).

After flushing with saline, the defect was filled with bone wax. The eight-plate was implanted in the contralateral side of the same limb in the Hemi-Epiphysiodesis technique group. Care was taken to avoid damage to the periosteum. With the aid of a full-size limb model, the tension band plate with two cannulated screws was inserted under the anteroposterior and lateral fluoroscopic guidance.

### Postoperative management

After surgery, an entire limb plaster was applied for 4 weeks to avoid a secondary fracture. Then, knee movement was permitted as tolerated by the patient. At postoperative week 8, the patient was allowed to place total weight on the leg. Radiograph data were obtained to evaluate the effect of the operation during follow-up.

### Statistical analysis

The Shapiro-Wilk was used for normal distribution analyses of the data. Data were presented as mean with min-max ranges for continuous variables and percentages for categorical variables. Data were analyzed by Chi-Squared Test using SPSS 22.0 software (SPSS Inc., Chicago, IL, USA). Statistically significant differences were considered when *P < 0.05*.

## Results

There were 16 (84%) patients who had an angular deformity > 10° and 5 (26%) patients with angular deformity > 20° before surgery. During the follow-up, 4 (21%) patients had an angular change of < 5°; 12 (63%) patients had angular deformity improvement > 5° averaging 10° (range, 5° to 23°), and 3 (16%) patients had increased of the angular deformity averaging 17° (range, 7° to 27°). There were 5 (26%) patients with angular deformity improvement > 10° in physeal bar resection combined with the Hemi-Epiphysiodesis group, while zero in the physeal bar resection group. There was a statistical difference between them (*P < 0.001*).

**Fig. 4:**
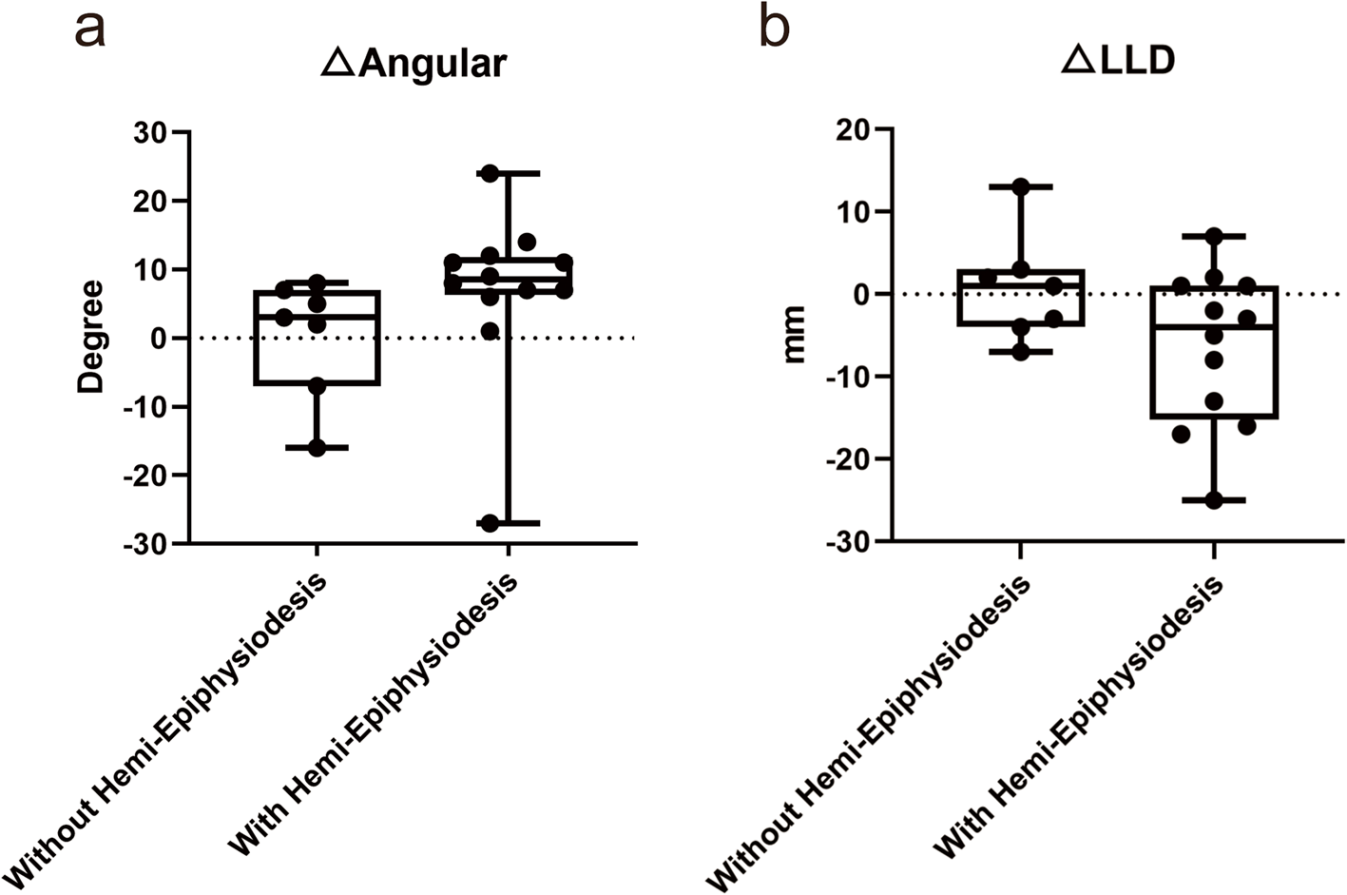
The Box-plots graphs of △Angular (**a**) and △LLD (**b**)

**Fig. 5:**
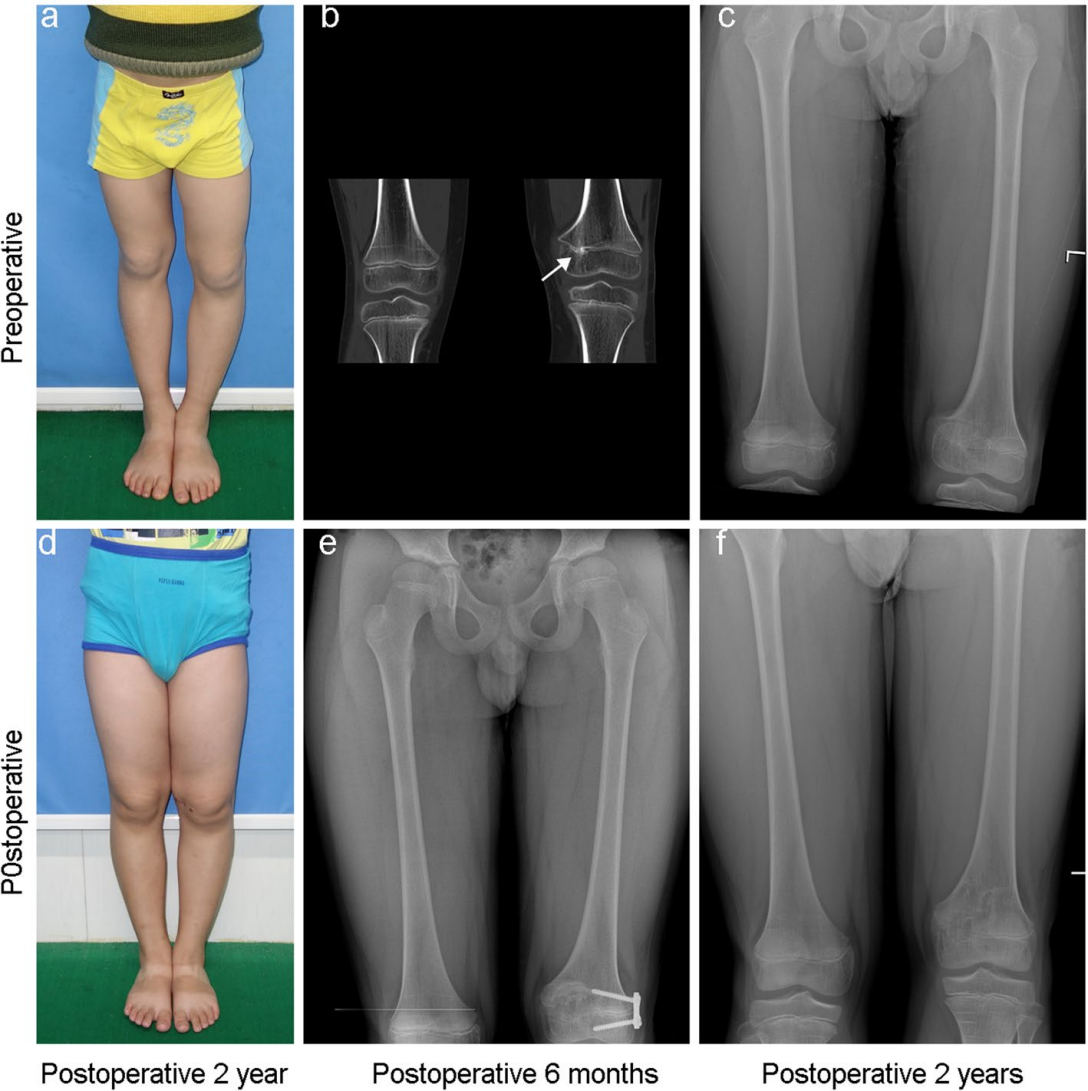
Typical case of bar resection (Case 10). The gross view (**a**), coronary CT (**b**), and anterior-posterior radiograph (**c**) of 9 years and 6 months old boy before surgery; (**d**) The gross view of the patient at postoperative two years; (**e**) The anterior-posterior radiograph of the patient at postoperative 6 months; (**f**) The anterior-posterior radiograph of the patient at two years

Before surgery, there were 12 (63%) patients with LLD > 10 mm, and 7 (37%) patients > 2 cm. All of the patients’ affected limbs were shorter than the control ones. After surgery, we found that 7 (37%) patients had a change of < 5 mm and were considered unchanged. Only 2 (15%) patients had LLD improvement > 5 mm, averaging 10 mm (range, 7 to 13 mm), and 7 (36.8%) patients became worse with LLD > 5 mm, averaging 13 mm (range, 5 to 25 mm). The MAD improved significantly in 11 patients (57.9%) with a correction distance of more than 10 mm, while the MAD became worse in 2 patients. The details are shown in Table 2 and Fig. 4. There were no postoperative fractures, infections, or intraoperative complications such as neurovascular injury. The typical case is shown in Fig. 5.

**Table 2:** Preoperative and postoperative deformity or shortening

Case	Angular deformity (degree)			△Angular	Mechanical axis deviation (mm)		LLD (mm)		△LLD
	Pre-op		Post-op		Pre-op	Post-op	Pre-op	Post-op	
1	17		24	-7	-42	-47	10	13	-3
2	7		4	3	-17	-15	14	21	-7
3	13		6	7	-23	-11	20	7	13
4	14		9	5	2	1	3	2	1
5	8		24	-16	0.9	0.3	4	8	-4
6	6		4	2	3.5	2.9	5	3	2
7	14		6	8	-17	10	21	18	3
8	27		3	24	-22	3	16	41	-25
9	36		15	11	-87	-13	25	30	-5
10	16		7	9	20	5	13	06	7
11	12		11	1	30	28	21	34	-13
12	25		18	7	48	33	34	42	-8
13	16		5	11	31	7	1	4	-3
14	15		8	7	33	12	3	5	-2
15	12		4	8	-10	1	22	21	1
16	23		11	12	-34	-10	8	6	2
17	18		4	14	4	4	6	5	1
18	22		16	6	45	33	19	35	-16
19	13	30		-27	32	56	22	39	-17

△*Angular* Pre-op angular – Post-op angular, △*LLD* Pre-op LLD – Post-op LLD

## Discussion

The decision for physeal bar resection combined with or without the Hemi-Epiphysiodesis technique is based on the patient’s age, remaining growth potential, location of injury within the epiphysis, size of physeal bar, the degree of limb deformity, an individual patient and family’s desire [10, 14]. Most patients with partial growth arrest secondary to trauma often develop some degree of angulation or limb-shortening deformity [15]. Still, as long as the physeal bar size is less than 50%, and there are two years or 2 cm growth remaining at the involved physis, the physeal bar resection can be helpful [16, 17]. This study found that most patients benefited from the physeal bar resection. Physeal bar resection combined with the Hemi-Epiphysiodesis procedure was more effective in correcting the angular deformity.

Osteotomy is the most common technique for correcting the angular deformity of a limb. At the same time, we commonly use Hemi-Epiphysiodesis for correcting angular limb deformities and sometimes for LLD in growing children [18]. Stevens reported an excellent result of Hemi-Epiphysiodesis for angular correction using tension band plates [19]. Physeal bar excision recovers the epiphysis growth ability but can’t regenerate the damaged epiphysis [20]. Hence, a Hemi-Epiphysiodesis technique based on the patient’s growth potential could play a critical role in correcting the deformity.

Physeal bar resection combined with the growth guide technique has been reported to treat partial growth arrest for a decade, while there are no definite indications established [21]. Fu et al. found that physeal bar resection and Hemi-epiphysiodesis are effective treatments for correcting ankle varus deformity [10]. Some surgeons suggested that osteotomy could be accepted when the correction of angular deformities > 20° [4]. Since they likely will not correct spontaneously after bridge resection. Williamson and Staheli recommended corrective osteotomy at the time of bridge resection for angular deformities > 10°, especially when the area of growth arrest is > 25% of the physis [7]. Though the osteotomy can simultaneously correct angular deformity and lengthen the short limb, a significant risk of recurrent deformity follows. Furthermore, the lengthening area may have delayed union or poor callus formation [12]. It is the last therapy to treat partial growth arrest when the remaining growth ability is limited. In this study, we found that most patients benefited from the physeal bar resection. In contrast, physeal bar resection combined with Hemi-Epiphysiodesis would be more effective in treating severe deformity. We thought the Hemi-epiphysiodesis procedure to treat the partial growth arrest is necessary when the angular deformity is > 10°.

The timing of Hemi-Epiphysiodesis is another issue worthy of careful consideration. Accurate Hemi-Epiphysiodesis timing is tricky because the growth disturbance varies depending on the physeal bar condition (area, location, and the remaining growth ability) [19, 22, 23]. Theoretically, the surgeon should remove the tethering device when the angular deformity has been corrected. However, the recurrence of deformation, the so-called rebound effect, is so common following the removal of the tethering device [18]. To avoid this, our department used Paley’s method to predict the potential growth ability [24]. For angular deformity, we favor delaying the removal of the tethering eight-plate until a small amount (about 5°) of overcorrection has occurred. If there is under-correction during the postoperative 2 years, the screws would be temporarily removed and reimplanted 6 months later.

There were some limitations to this study. First, this is a retrospective study, and the cases are small. Second, not all the patients were followed to maturity, and the time interval to obtaining postoperative imaging varied. Third, there was no data to assess the postoperative resecting bar area. Further studies are needed to reveal the effect of physeal bar resection combined with the Hemi-epiphysiodesis technique.

## Conclusion

Physeal bar resection combined with Hemi-epiphysiodesis is effective for partial epiphysis growth arrest. Without statistically verifying, we believe patients with limited growth ability could benefit more from physeal bar resection combined with Hemi-epiphysiodesis.

## Data Availability

The datasets generated and analyzed during the current study are available from the corresponding author upon reasonable request.
